# Psychometric properties of the Rowland Universal Dementia Assessment Scale – Peruvian Version among Indigenous Amazonian communities

**DOI:** 10.1038/s44400-026-00104-9

**Published:** 2026-08-14

**Authors:** Alicia Boluarte Carbajal, Arantxa N. Sanchez Boluarte, Marleny Nolasco, Monica M. Diaz

**Affiliations:** 1 School of Psychology, Universidad Cesar Vallejo, Lima, Peru; 2 Center for Global Health, Universidad Peruana Cayetano Heredia, Lima, Peru; 3 Department of Global Health, University of Washington, Seattle, USA; 4 Department of Neurology, University of North Carolina at Chapel Hill School of Medicine, Chapel Hill, USA

**Keywords:** Health care, Medical research, Psychology, Psychology

## Abstract

Cognitive screening tools are rarely validated for Indigenous populations of Latin America, a barrier to early dementia detection. We culturally adapted and evaluated the psychometric performance of the Peruvian version of the Rowland Universal Dementia Assessment Scale (RUDAS-PE) for Shawi Indigenous Amazonian communities. We included 434 adults aged ≥50 years who completed the RUDAS-PE. Cognitive interviews and community feedback revealed cultural mismatches, particularly in visuoconstruction tasks, requiring linguistic and contextual item modifications. The original 6-item structure showed poor fit, while a 5-item version demonstrated strong unidimensionality and improved model fit (confirmatory factor analysis, comparative fit index = 0.97; Root Mean Square Error of Approximation = 0.06). Item response analyses confirmed high measurement precision at low-moderate educational levels and identified severe floor effects in visuoconstruction. Culturally-valid adaptations of brief cognitive tools, particularly the visuoconstruction domain, are essential to avoid diagnostic bias in Indigenous populations. Our findings provide a scalable framework for adaptation of cognitive screening for Indigenous communities.

## Introduction

Dementia is one of the leading causes of disability in older adults and the seventh leading cause of death worldwide^1^. Currently, an estimated 55 million people worldwide live with dementia, making it an international public health priority^2^. In Latin America, the prevalence of dementia is 8.4%, which is comparable to or higher than estimates of 7–8% in high-income countries^3^, while in Indigenous communities it appears to be relatively low, with estimates of 0.9–4.9%, particularly in Amazonian populations in Brazil and Bolivia^4,5^. However, the scarcity of epidemiological studies and the lack of instruments to detect dementia make it particularly difficult to study.

In recent years, the study of cognitive impairment in Indigenous populations has become particularly relevant due to the need to understand how sociocultural, environmental, and biological factors affect the brain aging process^6^. This perspective has driven the development and adaptation of psychological assessment tools that enable the early identification of cognitive decline and, therefore, improve the timely detection of dementia^7,8^. Native communities generally have lifestyles, linguistic patterns, and traditional knowledge systems that can differ significantly from the urban contexts in which most cognitive assessment instruments have been developed. Therefore, it is crucial to design and adapt tools that account for unique aspects of these populations, the context in which they live, and their daily experiences.

Historically, Indigenous peoples have been systematically marginalized from their rights and decisions that directly affect them, leading to the loss of their lands, their culture, limited access to resources and services, and food insecurity^9,10^. The Shawi Indigenous people live mainly in the districts of Cahuapanas and Balsapuerto (Loreto region) in the Amazon, and are considered one of the most vulnerable Amazonian groups, with 26,841 inhabitants^9,10^. It is estimated that 8% of Shawi people in Loreto, Peru, are over 50 years old, based on 2017 National Census data^11^. This results in approximately 2126 Shawi people over the age of 50. The Shawi people live on a subsistence economy consisting of agriculture, fishing, and hunting^12^. They are also the Peruvian ethnic group with the lowest percentage of state recognition of their lands and institutional affiliation^13^. This reality shows that Indigenous peoples face poorer economic, social, and health conditions as a result of structural inequalities^10,14^. Thus, the cognitive function assessment in Indigenous populations requires culturally sensitive approaches, without biases accounting for language, culture, literacy, and educational level^15^.

Specifically, the Rowland Universal Dementia Assessment Scale (RUDAS) has been increasingly studied as a culturally valid alternative to traditional cognitive screening tools^16^ used in various multicultural contexts as a screening tool^17–20^. It has been shown to have comparable or superior performance to the Mini Mental State Exam (MMSE) in the detection of mild cognitive impairment and dementia, particularly in people with low educational attainment or illiteracy^21,22^. The RUDAS has been shown to have good concurrent validity^20–24^, but few studies report evidence of construct validity. Those that do report construct validity have been conducted with small samples, limiting its analysis^25,26^. It is crucial that cognitive screening tests reflect robust psychometric properties of validity and reliability, particularly when adapted to culturally and linguistically diverse populations.

Our study represents the first attempt to analyze the psychometric performance of RUDAS in older adults from Shawi Amazonian communities. We sought to examine the validity and reliability of the RUDAS in this population. Following cross-cultural adaptation, our study used modern psychometric frameworks examining structural models through Classical Test Theory and Item Response Theory to explore the latent structure of cognitive domains and refine measurement accuracy of the Peruvian version of RUDAS^27^.

## Results

### Cultural adaptation

Through meetings with representatives of a sample of selected communities, we explored their worldview, values, beliefs, and community practices, which served as input for the next stage of cultural adaptation.

Eight cognitive interviews were conducted with people over the age of 50 in various social roles in their community (such as healers, “Apus,” elders, and bilingual teachers), which helped identify how the Shawis conceptualize concepts such as attention, memory, and neurological health. Key aspects were identified, such as the perception of time, traditional forms of knowledge transmission, and the conceptualization of memory for remembering recent events. Sociocultural and linguistic differences were found. These findings allowed us to incorporate concepts related to their traditional activities, ensuring that the items and each domain reflected the cognitive profile of the Shawi people.

The RUDAS-PE adaptations prioritized familiarity with specific tasks based on the local ecological context. In Component 1, which assesses immediate memory, the word “COFFEE” was replaced with “RICE,” as it is a commonly consumed and locally cultivated food in the surrounding districts. With respect to the visuoconstruction component, difficulties in completing the drawing task were identified; nevertheless, it was considered appropriate to retain it. Other changes were made in the Judgment component. The three original items were replaced with four items designed based on participants’ daily activities, social organization, and Amazonian worldview:

1. Item: “Why do you think some people live longer than others?” aimed at assessing reasoning.
2. Item: “If you arrive in a new community, how would you locate a friend you want to see?” replacing an urban context with a community-based context.
3. Item: “Tell me, what roles does an ‘Apu’ fulfill in the communities?” assessing knowledge of their social context and comprehension.
4. “How is a communal work activity organized?” assessing sequencing and planning.

We adjusted the wording and scoring of the test items using the prior suggestions. Translation and back-translation into the original language ensured semantic equivalence and eliminated cultural biases, incorporating relevant elements of the Shawi community’s sociocultural context.

During the pilot study, comprehension problems, instruction, and item ambiguities were resolved, and test administration time was adjusted. These adjustments allowed the test to become a culturally contextualized dialogue, reducing administration time by minimizing participants’ resistance to tasks that are unfamiliar to their daily lives. Translation and back-translation into the original language ensured semantic equivalence and reduced cultural bias, while incorporating relevant elements of the Shawi community’s sociocultural context.

During the pilot study, issues related to comprehension, ambiguities in instructions, and items were resolved, and the test administration time was refined.

### Descriptive analysis

Sociodemographic and medical characteristics of enrolled participants are summarized in Table 1. Most participants (66.4%) did not have any formal education, and at most 11.1% of our participants completed primary school. Only 14.9% of participants knew how to use a pencil.

**Table 1:** Sociodemographic and clinical characteristics of participants (*N* = 434)

Characteristics *	*n* (%), median (IQR), mean (SD)
Sociodemographics **	
Age (years)	59 (54–67)
Female sex	232 (53.5)
Years of education	0 (0–3)
Civil status	
Single	30 (6.9)
Married/Cohabitating	310 (71.4)
Widowed	80 (18.4)
Salaried job	13 (2.9)
Electricity	278 (64.1)
Internet	31 (7.1)
N people in same household	4 (3–6)
Shawi ethnicity	380 (87.6)
Monolingual Shawi	271 (62.4)
Bilingual Shawi - Spanish	107 (24.7)
Daily traditional alcohol intake	396 (91.2)
Clinical ***	
BMI (kg/m^2^)	22.6 (20.7–24.5)
Systolic blood pressure (mmHg) †	120 (108.8–130)
Diastolic blood pressure (mmHg) †	72 (65–80)
Random glucose (mg/dL)	111 (103–123.8)

*Percentages calculated based on number of participants included in the analysis (*n* = 434).

**Age had 6 NAs; sex, 4 NAs; remaining sociodemographic variables, 10 NAs.

***BMI had 41 NAs; blood pressure, 10 NAs; glucose, 36 NAs; previous diagnosis, 11 NAs.

†Blood pressure measured in the right arm.

**Fig. 1:**
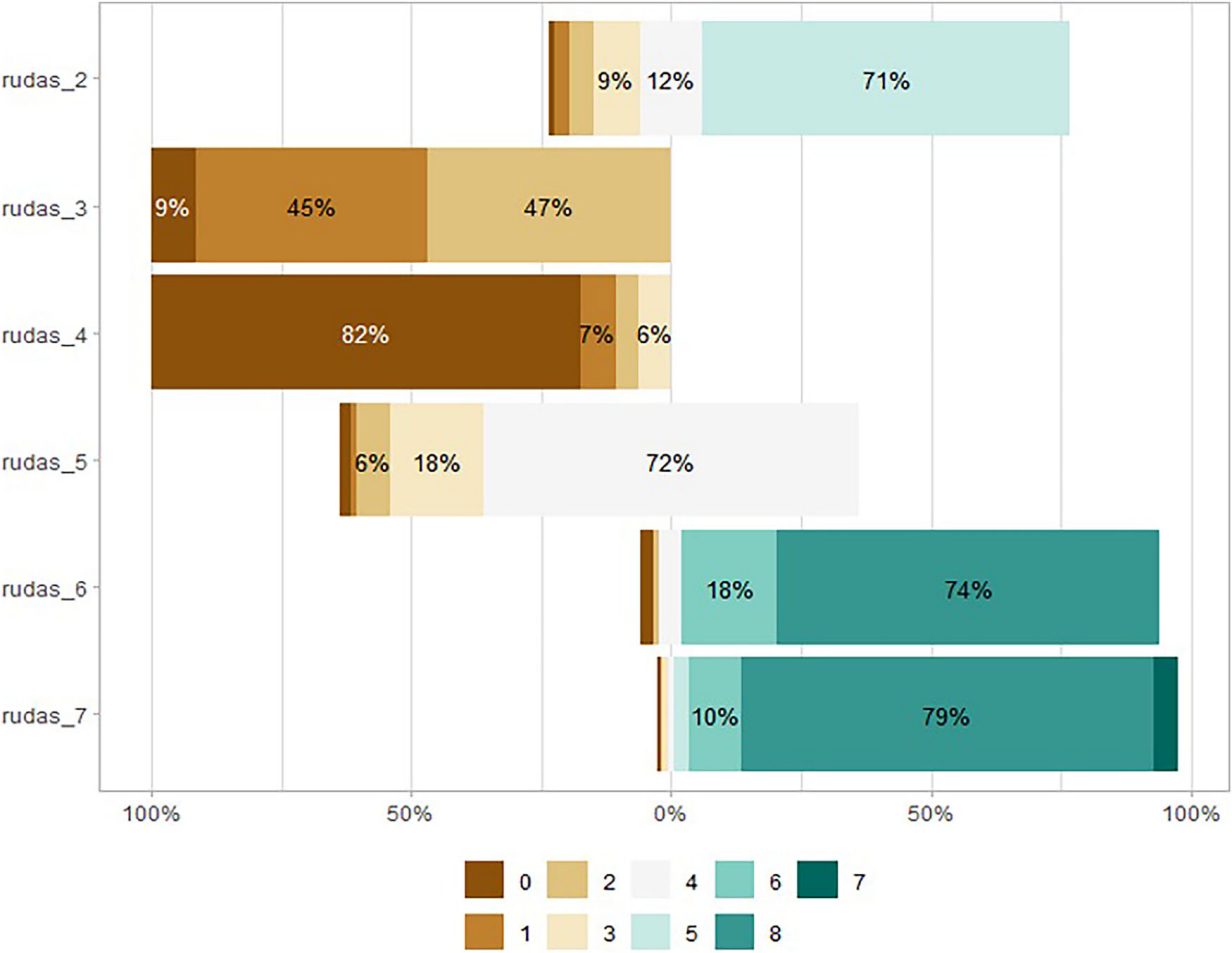
Visualization of RUDAS-PE response ratios.

Figure 1 details the proportional distribution of responses for each of the six dimensions of the RUDAS-PE scale. As expected in a cognitive screening scale applied to a community sample, most items demonstrated an asymmetric distribution with a pronounced ceiling effect. Specifically, items 2 (Orientation), 5 (Judgment), 6 (Memory), and 7 (Language) showed that between 71% and 79% of those evaluated obtained the maximum score. This pattern is consistent with a population largely without cognitive impairment. However, item 4 (Visuospatial Construction) exhibited markedly anomalous behavior, showing a severe floor effect: 82% of the sample obtained the minimum score of 0. This low variability and its distribution, which is opposite to that of the rest of the scale, suggest that item 4 may not be functioning consistently with the other dimensions (Fig. 1).

**Fig. 2:**
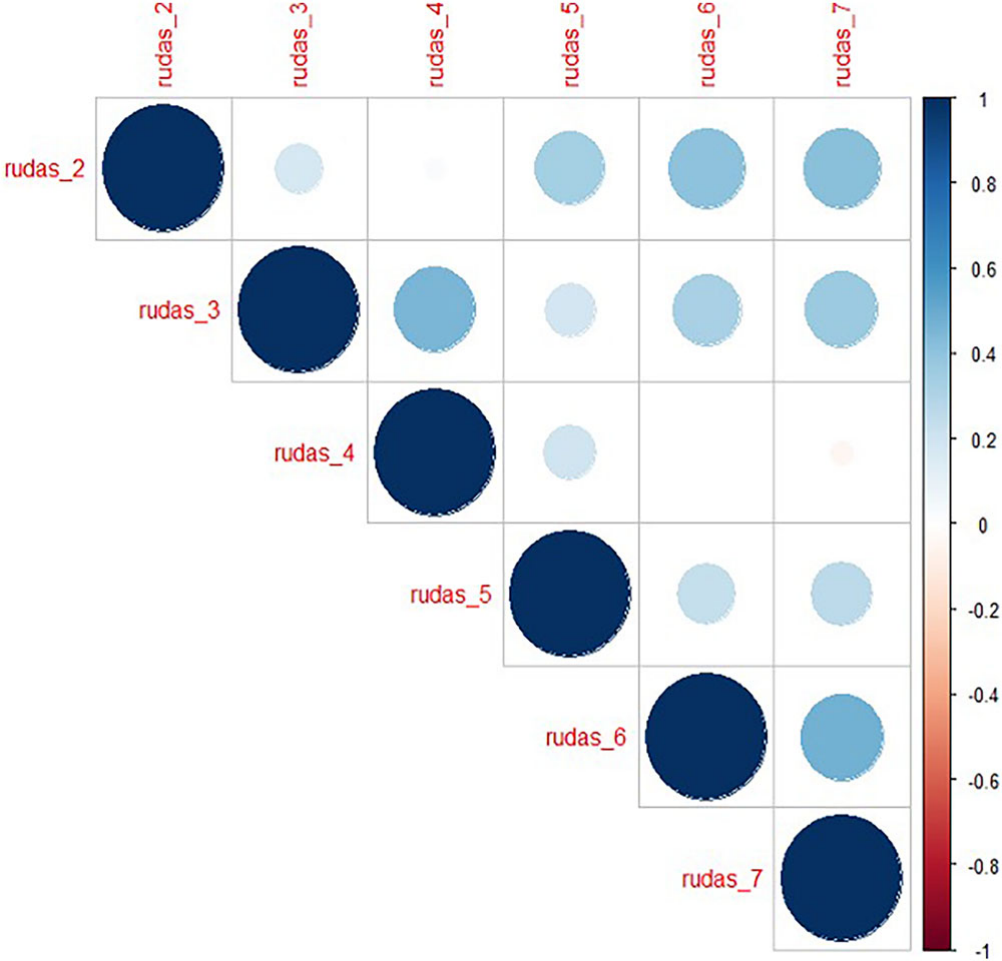
Polychoric correlations between RUDAS-PE items.

To examine the interrelationship between the dimensions of the RUDAS-PE and to evaluate the internal consistency of the scale, a polychoric correlation matrix was calculated, the results of which are shown in Fig. 2. The analysis revealed moderate and statistically significant positive correlations between most items, with coefficients ranging from 0.19 to 0.48, suggesting that they share a common latent construct.

However, in line with the anomalies detected in the descriptive analysis, item 4 (Visuospatial Construction) shows a markedly divergent correlation pattern. This item shows correlations close to zero and insignificant with virtually all other dimensions of the scale. This lack of consistency, probably exacerbated by the severe floor effect previously observed, compromises its construct validity and suggests that its inclusion in a one-dimensional model would negatively affect the overall fit, justifying its potential exclusion in the factorial modeling stage.

### Confirmatory Factor Analysis

To evaluate the structural validity and test the unidimensionality hypothesis of the RUDAS-PE scale, two Confirmatory Factor Analysis (CFA) models were fitted. The first model (M1) included the six original dimensions, while the second model (M2) was re-specified excluding item 4, whose anomalous behavior was identified in the descriptive and correlation analyses.

Table 2 summarizes the fit indices for both models, demonstrating a substantial and decisive improvement in the re-specified model. Model M1 showed a poor fit to the data, with a significant Chi-square value (χ²(9) = 46.306, *p* < 0.001), a CFI of 0.884, and a TLI of 0.807, both below acceptable thresholds. In addition, the RMSEA was high (0.099), indicating a considerable discrepancy between the model and the observed data. In stark contrast, model M2 showed excellent fit: although the Chi-square remained significant (χ²(5) = 12.887, *p* = 0.024), the relative fit indices improved dramatically to a CFI of 0.969 and a TLI of 0.939. The RMSEA was also reduced to 0.061. This remarkable improvement in all fit indices confirms that the exclusion of item 4 resolves the main source of misfit and supports the plausibility of a unidimensional structure for the five-item scale.

**Table 2:** Adjustment indices between the two CFA models from RUDAS-PE

Model	X^2^ (gl)	p (Chi2)	CFI	TLI	RMSEA	RMSEA CI	SRMR
M1	46.306 (9)	< 0.001	0.884	0.807	0.099	[0.072, 0.128]	0.097
M2	12.887 (5)	0.024	0.969	0.939	0.061	[0.020, 0.103]	0.047

IRT models from RUDAS-PE

### IRT Model: Generalized Partial Credit Model

Following the same model specification logic used in CFA, the performance of the RUDAS-PE scale was evaluated under the IRT framework. A Generalized Partial Credit Model (GPCM) was fitted to examine the fit of the data to a unidimensional structure. Table 3 presents the fit indices for the two IRT models: the initial model (M1) with six items, and the re-specified model (M2) with five items, excluding rudas_4. The results of the IRT analysis strongly corroborate the CFA findings. Although model M1 shows a marginally acceptable fit (CFI = 0.941, TLI = 0.902), it has an RMSEA (0.074) that suggests room for improvement and a significant Chi-square value (*p* < 0.001). In contrast, model M2 shows a dramatic improvement and excellent fit to the data, with a non-significant Chi-square (*p* = 0.303), CFI and TLI indices close to unity (0.997 and 0.993, respectively), and a very low RMSEA (0.022). This convergence of evidence between the CFA and IRT frameworks reinforces the conclusion that the five-item model is psychometrically superior and more robust.

**Table 3:** Adjustment indices between the two

GPCM-IRT models from RUDAS-PE Model	X^2^ (gl)	p (Chi2)	CFI	TLI	RMSEA	RMSEA CI	SRMR
M1	29.689	0.000	0.941	0.902	0.074	[0.045, 0.104]	0.077
M2	6.037	0.303	0.997	0.993	0.022	[0.000, 0.074]	0.067

**Fig. 3:**
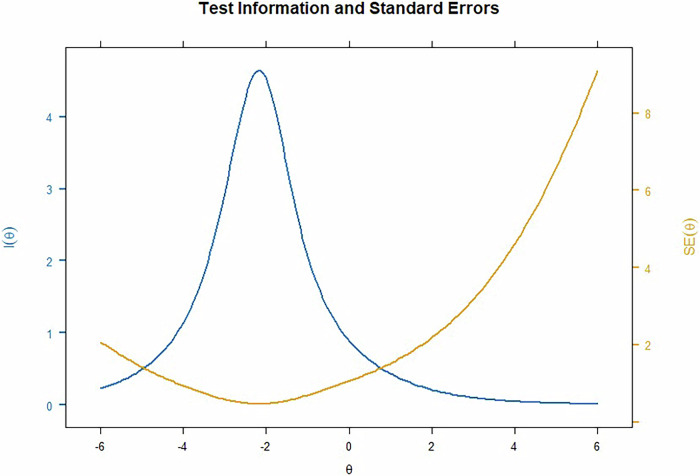
Test Information and Standard Errors.

Figure 3 shows the Test Information Curve (TIC) and its corresponding Standard Error of Measurement (SEM) curve. The ITC, which is the sum of the information from the individual items, reaches its peak at a skill level of approximately theta = −2.0. This confirms that the five-item RUDAS-PE scale, as a whole, offers the highest measurement accuracy for identifying individuals on the low-moderate cognitive ability spectrum. Conversely, the SEM curve shows that measurement error is minimal in this same range and increases progressively as ability moves away from this optimal point, especially at higher ability levels (theta > 1), where the test loses its discriminatory power.

**Fig. 4:**
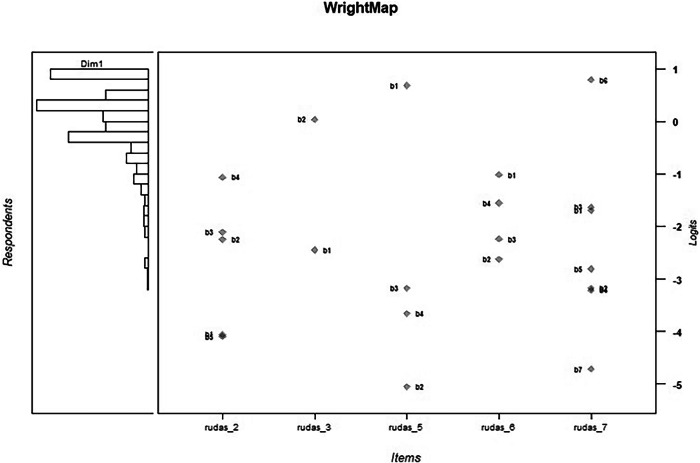
Wrights Map for the distribution of items.

Figure 4 (Wright Map) provides an integrated visual representation that aligns the distribution of participants’ ability (histogram on the left) with the distribution of item threshold difficulty (dots on the right) on the same logit scale. The map visually confirms the conclusion derived from the Test Information Curve. There is a partial mismatch between the difficulty of the test and the ability of the sample. Most of the difficulty thresholds of the items are located in the low to moderate ability range (theta from −5.0 to −1.0), while most participants are grouped at a higher ability level. This indicates that the test, in its current form, is more difficult than necessary for most test-takers and is better targeted to accurately differentiate between individuals at the lower end of the cognitive spectrum.

**Fig. 5:**
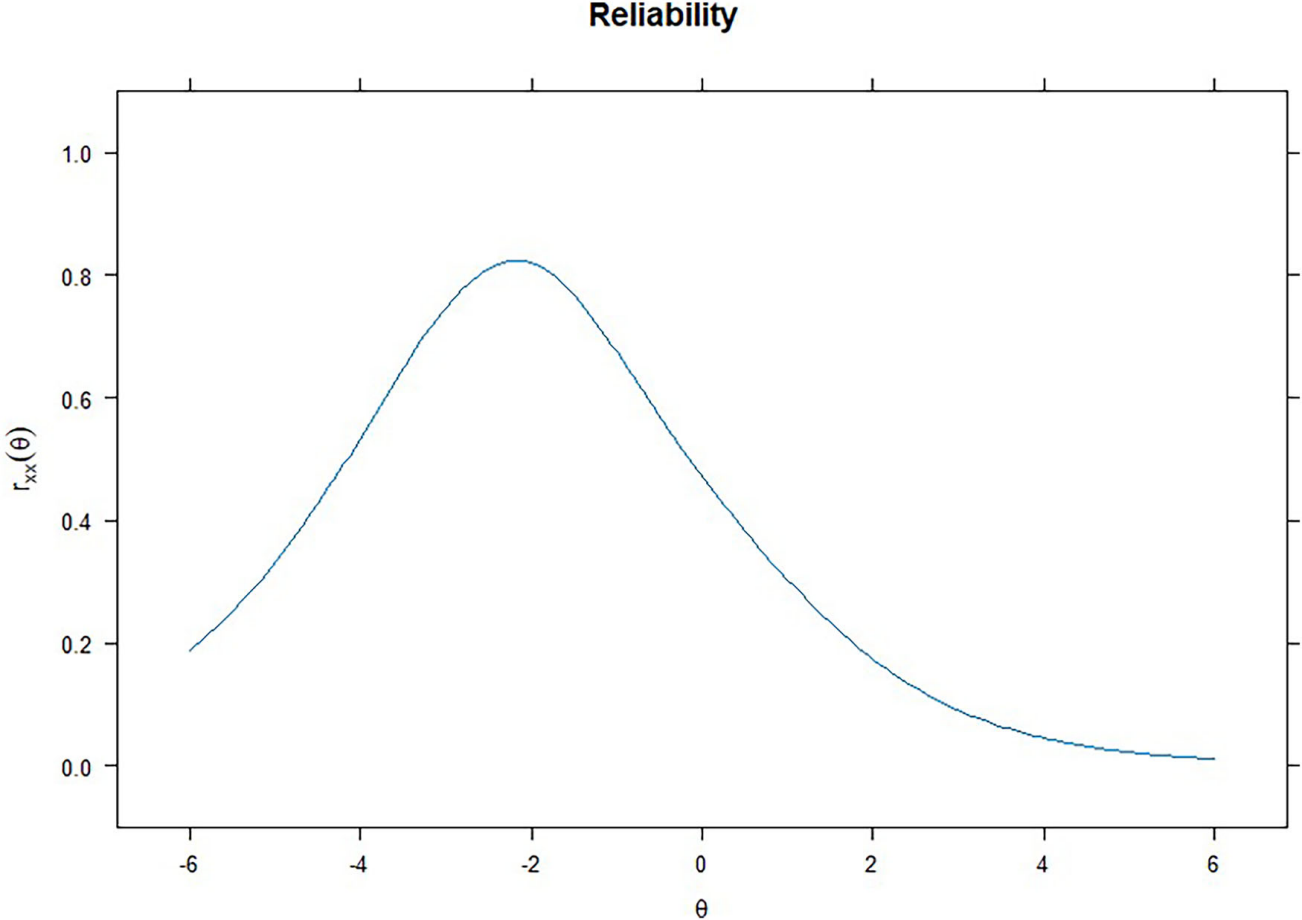
Reliability of adapted RUDAS-PE.

Finally, Fig. 5 shows the reliability function of the test, which varies depending on the skill level (theta). In line with the Test Information Curve, reliability peaks (at approximately 0.83) at a skill level of theta ≈ −2.0, the same point where the test provides the most information. Reliability decreases as participants’ ability moves away from this optimal point, being considerably lower in the high ability ranges. These findings highlight one of the conceptual advantages of IRT over CTT: reliability is not a static property of the test, but a function of the interaction between test difficulty and individual ability. For this sample, the marginal (average) reliability of the test was 0.48, a modest value that reflects the mismatch between the difficulty of the test and the skill distribution of the population assessed.

### Complementary analysis of education and RUDAS-PE items

We examined the association between years of education and RUDAS-PE performance (*ρ* = 0.31, *p* < 0.001). Spearman correlations between years of education and item scores varied across domains. The strongest association was observed for item 4 (visuoconstruction; *ρ* = 0.43, *p* < 0.001). Other items showed weak correlations ranging from *ρ* = 0.11 to 0.21, while item 6 was not significantly associated with education (*ρ* = 0.02, *p* = 0.74). When item 4 was excluded from the total score, the correlation between education and overall performance decreased (*ρ* = 0.22, *p* < 0.001).

## Discussion

Analyzing the validity and reliability of the RUDAS-PE represents a significant advance in the assessment of individuals from Indigenous communities, allowing for more accurate and culturally valid evaluations of cognitive function. This effort has important implications for its clinical use in screening individuals for cognitive impairment to determine who is at highest-risk and may need additional testing or clinical assessment.

By integrating qualitative and quantitative approaches, we sought to ensure that the instrument accurately captured the cognitive construct relevant to this cultural context. Although RUDAS was designed as a screening tool for culturally, linguistically, and educationally diverse populations^16^, other studies have shown that adaptations are still needed to accurately assess dementia in diverse populations^28,29^.

The RUDAS-PE was previously adapted to the context of Peruvian population^22^. However, our cross-cultural adaptation process showed that it could not be used to identify cognitive deficits without linguistic, semantic, and cultural modification; its direct use in a culturally different population could lead to misclassification rather than true cognitive impairment.

Despite the adverse socioeconomic conditions of this community, the natives of the Shawi ethnic group still preserve the vitality and regional diversity of their language. This provided key cultural considerations for the study, given the impact of language on the understanding of their social, cultural, and health reality. In addition, we found that Shawi communities had ecological knowledge with unique linguistic structures and daily practices that differed substantially from those assumed in standardized instruments. These contextual differences highlight that culturally-based adaptation is essential not only to improve measurement accuracy but also to promote health equity in dementia screening in underserved populations^30^.

The cross-cultural adaptation process strengthened the content and apparent validity of the tool and ensured that its administration is appropriate, understandable, and culturally responsive. Cognitive interviews were used to modify and add culturally relevant items, especially in the memory and judgment function. Other studies conducted in diverse populations agree with the use of cognitive interviews to detect possible comprehension difficulties focused on the user^31^, unlike content validity, which was not used in the present study, as it is oriented toward the review of items by expert opinion^32^. These cultural adaptation procedures in screening tests are supported by the scientific literature to improve the reliability of the information collected^33,34^.

In our descriptive analysis of the items, a ceiling effect is observed in most items, with 71% and 79% showing a maximum score. This response pattern reflects a ceiling effect similar to the findings reported in other screening tests^27^, that are not commonly reported, demonstrating a statistical and methodological weakness.

Item 4, corresponding to the visuoconstruction function, showed a severe floor effect and no correlation with other items. This anomalous behavior is consistent with a study conducted in Nepal in which difficulty understanding the visuoconstruction item was the most challenging aspect among a majority of uneducated participants^19^. Similarly, one study reported a ceiling effect in 75% of the sample and also showed that item 4 was associated with schooling^35^. In several countries, it has been shown that RUDAS results are independent of educational level and linguistic properties, highlighting RUDAS as an appropriate tool for populations with low educational levels^15,16,20,22^. However, in our sample, characterized by low or no formal education, the visuoconstruction domain, a skill previously associated with formal schooling, showed limited performance, which adds a point of controversy to the existing literature^36^.

This low performance on item 4 probably reflects a cultural incongruity rather than true cognitive impairment due to contextual differences. This pattern was also observed in studies that adapted visuospatial tasks for Australian populations^37^. One possible explanation is that educational exposure influences familiarity with the format and demands of cognitive testing rather than underlying cognitive ability. These results indicate that the relationship between schooling and performance on the RUDAS is not uniform across all populations and reinforces the need to obtain validity evidence in different sociocultural contexts.

Our findings indicate that RUDAS-PE total scores were influenced by years of formal education. This effect was driven primarily by the visuoconstruction task (item 4), which showed the strongest association, contributing disproportionately to the relationship between RUDAS performance and educational level in this population. Besides, 82% of participants scored zero to item 4, of whom 93% did not know how to use a pencil, and 75.3% did not receive any formal education. Moreover, the majority of participants were monolingual in Shawi and had limited access to digital resources, such as the internet, which may influence linguistic flexibility, exposure to standardized concepts, and interaction with abstract tasks, which may further impact the effect of education on performance on the RUDAS-PE.

This suggest that the relationship between education and cognitive screening outcomes is mediated by sociocultural context, and, particularly, that item 4 may reduce the cultural fairness of the RUDAS-PE in highly marginalized, low-literacy settings. This could have implications for clinical interpretation of cognitive screening, as misclassification may occur if educational and cultural factors are not adequately considered in Indigenous populations.

Most studies have confirmed the diagnostic accuracy of the RUDAS through sensitivity and specificity analyses with AUC values exceeding 0.90^22,23,38,39^. However, there are few studies based on the validity of the internal structure, which can be a prerequisite for establishing criterion validity^40–42^. Our findings demonstrate a unidimensional structure and a substantially superior fit after removing item 4, similar to a study conducted in Indonesia demonstrating unidimensionality of RUDAS^42^.

Both the CFA and IRT support the effectiveness of using a 5-factor scale. Likewise, Rasch analysis revealed that the five-item M2 offers greater measurement accuracy at low to moderate cognitive ability levels (θ = –2) (Fig. 3). This suggests that the abbreviated version of the RUDAS-PE is adequate for detecting deficits in people with low cognitive functioning, but loses discriminatory power as response ability increases, showing a discrepancy between the ability of the people assessed and the difficulty of the test (Fig. 4). A similar situation was found in the Rasch analysis performed with the Montreal Cognitive Assessment (MoCA), where easy items showed low levels of discrimination, reducing measurement accuracy in high-functioning groups. Another study performed a Rasch-based analysis of the MMSE and found that the person’s ability was greater than the difficulty of the item^43^.

An important finding is the limited performance of the visuoconstruction domain (Item 4). Despite the theoretical relevance of visuoconstruction, its performance in this population was characterized by a severe floor effect and lack of correlation with other items, suggesting that it did not function as a valid indicator of cognitive ability among the Shawi. Item-level analyses further suggest that item 4 did not function as a marker of cognitive impairment, but was strongly influenced by educational exposure among the Shawi population. The literature shows that the visuoconstruction domain is the one most strongly correlated with educational level^19,35,36^, in agreement with our data.

The resulting 5-item version of the RUDAS-PE demonstrated strong unidimensionality, supporting the interpretation that the domains of memory, language, executive functions (judgment), and perceptual-motor functions (praxis and orientation) capture a common latent construct that can be interpreted as a measure of Global Cognitive Functioning (GCF)^35^, consistent with the original structure of the RUDAS instrument^16^. In the Shawi context, GCF manifests as a measure of cognitive integrity with high ecological validity, prioritizing functional domains essential for the autonomy of older Indigenous adults over technical skills dependent on formal schooling.

Moreover, this study should not be interpreted as a validation of the original RUDAS cognitive screening tool, but rather as the development of a culturally adapted and psychometrically robust version of the RUDAS-PE for use in Shawi Indigenous populations. Our study represents one of the first applications of Rasch modeling to RUDAS, providing a novel contribution to the psychometric evaluation of this widely used cognitive screening tool. We highlight the need to revise or supplement the item pool to improve coverage across the cognitive spectrum, and believe research efforts are needed to develop a culturally sensitive visuoconstruction task that does not involve using a pencil.

For reliability (Fig. 5), our results reveal particular patterns. Reliability based on the Rasch model peaked (0.83; *θ* ≈ −2.0), indicating that the instrument provides greater reliability at low levels of cognitive ability. This magnitude is consistent with the internal consistency coefficients documented in various international validation studies conducted in Ethiopia *α* ≥ 0.73, Nepal *α* ≥ 0.70, Brazil *α* ≥ 0.69, and in patients with traumatic brain injury, alpha coefficients between *α* ≥ 0.69 and *α* ≥ 0.74 have been reported^18–20,44^. In Peru, a study found an alpha of *α* ≥ 0.65 in older adults with low educational levels^23^. However, the marginal reliability (*ρ* = 0.48) shows little accuracy for people with high cognitive functioning, suggesting that internal consistency may vary according to the subject’s ability.

An important consideration in interpreting these findings is the distinction between marginal and conditional reliability. Although the marginal reliability of the instrument was modest (*ρ* = 0.48), reflecting limited precision across the full range of cognitive abilities, the test demonstrated substantially higher reliability (0.83) at lower levels of cognitive ability (*θ* = –2.0). This pattern indicates that the instrument is most precise in the range where screening for cognitive impairment is most needed and that our adapted version of the RUDAS-PE is better suited for identifying individuals with low to moderate cognitive ability. Therefore, classification accuracy is expected to be highest among individuals at risk for cognitive impairment, but lower among cognitively healthy individuals.

These findings support the use of the RUDAS-PE as a population-level screening tool in underserved and low-education settings with limited access to specialized care, where the primary goal is to identify individuals who may require further evaluation. The instrument should not be used in isolation for individual-level diagnostic decision-making, and results should be interpreted in conjunction with clinical assessment and additional testing when available. By cross-culturally modifying the items, potential cultural bias was reduced, and the ecological validity of the instrument was improved. This study ensures that cognitive screening is both linguistically and culturally appropriate for a historically underserved population. Accurate screening for cognitive impairment risk in these communities is crucial for reducing health disparities. Furthermore, item-level analysis using the Rasch model demonstrates a rigorous approach to tool validation beyond classical methods, setting a precedent for future adaptations of cognitive assessments in diverse cultural contexts^27^. Combining cultural adaptation with modern psychometric techniques to improve brain health is key, as it ensures that screening instruments detect risk without measurement bias that could lead to misclassification in Amazonian peoples.

One of the major limitations of the study was the linguistic barrier between the research team and the Shawi language, as most participants were monolingual Shawi speakers. This required our team to rely on Shawi-Spanish interpreters, which may have introduced variability in test administration despite efforts to standardize procedures. Furthermore, geographical accessibility and climate conditions posed logistical challenges affecting data collection in this remote setting. Another limitation is that we did not report the diagnostic accuracy of the tool, since we prioritized cross-cultural adaptation and evidence of structural validity as a prerequisite to criterion validity (sensitivity and specificity). Future studies are needed to develop a culturally appropriate visuoconstruction task and to evaluate the diagnostic performance of the RUDAS-PE against established clinical criteria in this population.

Overall, this study provides preliminary evidence that the culturally adapted 5-item RUDAS-PE test meets psychometric criteria for screening and detection of cognitive impairment in Indigenous communities. The instrument demonstrated strong structural validity and acceptable reliability for screening purposes. Both CFA and IRT analyses supported a unidimensional convergent model after exclusion of Item 4, given the stability of its scores, coherence, and adequate factor loadings and correlations among items. The marginal reliability (0.48) indicates limited precision and discrimination in individuals with higher cognitive performance, while the conditional reliability (0.83) suggests good measurement precision in identifying those at risk, consistent with the test information curve showing optimal performance in the low-to-moderate range of cognitive function. This findings support its potential utility as a screening tool for identifying individuals at risk of cognitive impairment, as an initial step for detection and timely referral.

This work expands the psychometric evidence for RUDAS in contexts of low educational attainment and Indigenous languages. Moreover, our findings highlight that psychometric properties may vary substantially across contexts, and that item-level evaluation is critical to ensure that instruments measure the intended construct rather than external factors such as educational exposure or test familiarity. This research acknowledges the importance of conducting cross-cultural adaptations and adhering to the methodology required to obtain evidence of validity of an instrument.

Finally, the adapted 5-item RUDAS-PE in the Shawi Indigenous community represents a meaningful step toward improving access to a community-based screening tool in underserved Indigenous populations, supporting the development of public policies to reduce brain health disparities. By providing a culturally grounded tool with demonstrated structural validity and precision in lower cognitive ranges, it may facilitate early identification of individuals at risk for cognitive impairment and support timely referral for further assessment and specialized care^45^. Furthermore, this study expands research perspectives in historically underrepresented populations, providing a foundation for future studies and establishing a precedent for the detection and assessment of cognitive impairment risk among Indigenous people in the Peruvian Amazon.

## Methods

### Design

We conducted a psychometric study that aimed to ensure comprehensive and rigorous assessment of instrument reliability and validity^46^. We conducted analyses using Classical Test Theory (CTT) and Item Response Theory (IRT). Qualitative and quantitative methods were used aligning with international standards for test adaptation^41^.

### Participants

Participants were recruited between October 2024 and February 2025 in rural communities recognized as Shawi by the Ministry of Culture, in the district of Balsapuerto, in the Loreto region of Peru. This study included adults over 50 years of age who met at least one of the following criteria: 1) native language of Shawi, 2) residence in the district of Balsapuerto, either in its town or across the Shawi communities and speaking Shawi and/or Spanish. Participants were recruited using door-to-door visits to the home and community meetings, strategies that were necessary given the scattered geography and adverse weather conditions in the area. Recruitment was also conducted during the monthly government programs, such as “Pensión 65” and “Juntos”, when Peruvian citizens (including Shawi) over age 65 receive a monthly incentive from the government.

### Procedures

We first adapted the RUDAS-PE was adapted using qualitative methods. First, opportunities for dialogue were created with residents and community leaders of the Shawi community, allowing for a deep immersion into Shawi culture. These informal group meetings were part of an apparent validity process^47^, in which interviews were conducted with two community leaders and two Shawi residents, as well as local government leaders. We administered the RUDAS-PE during each interview and collected any observations and feedback on the RUDAS-PE items. Using this process, we assessed acceptability and understanding of each instrument item. Subsequently, we conducted cognitive interviews to these participants to obtain evidence of validity based on the response process^48^, to ensure the instrument was understandable and relevant to the target population.

We then incorporated feedback from these cognitive interviews. Native experts from the Shawi culture (“Apus,” primary school teachers in the community) acted as bilingual (Spanish/Shawi) facilitators to incorporate the feedback we received to adapt the instrument. Finally, we conducted iterative pilot tests to evaluate the suitability of each item translated and back-translated by two native Shawi speakers. The objective of this process was to ensure the semantic, conceptual, and cultural adequacy of the instrument to ensure that the instructions and items were understandable and relevant to the Shawi participants.

Following the pilot testing, data collection was conducted. The data collection team was comprised of two native Shawi speakers with higher technical training, a Shawi teacher, a medical graduate, and three researchers, who participated on an ad hoc basis.

Permission to access 36 native communities was obtained through the “Apus,” who are the authorities representing each community. The evaluations were carried out through home visits and meetings and people voluntarily agreed to participate by signing the Informed Consent form. Nearly all of older adults in each selected community were evaluated.

### Instruments

The RUDAS is a brief six-item test that assesses memory, praxis, language, judgment, visuoconstruction, and body orientation^16^. It was originally developed in a multiethnic Australian population, where diagnostic accuracy did not vary by years of education (*p* = 0.20) or preferred language (*p* = 0.33). The RUDAS showed a sensitivity of 89% (95% CI: 76%–96%) and a specificity of 98% (95% CI: 88%-97%) with a cutoff point of 22/23 out of 30. The scale has been translated and adapted into multiple languages, although not all versions have undergone formal validation and diagnostic accuracy studies^49^.

In Peru, the RUDAS has been studied in Spanish-speaking urban and rural populations and demonstrated concurrent validity and its ability to discriminate between dementia and mild cognitive impairment^22,23,38,39^. However, some studies reported alpha coefficients below the expected optimal values^22,23,38^.

This study used the Peruvian version of the RUDAS (RUDAS-PE), linguistically and culturally adapted for use in Peruvian populations and is considered the most appropriate version for populations with sociocultural diversity and low levels of education^38^.

### Data Analysis

Among the total number of participants (*n* = 472), we excluded those without RUDAS-PE cognitive testing and remained with 434 participants for the purpose of this analysis. Initially, an exploratory analysis of the data was performed to understand the behavior of each of the six dimensions that make up the RUDAS-PE. The response ratio for each score category was reported in order to identify possible floor effects (accumulation of responses in the lowest categories) or ceiling effects (accumulation in the highest categories), which could limit an item’s ability to discriminate between participants^50^. Given the ordinal nature of the dimension scores, a polychoric correlation matrix was estimated for the classical test theory (CTT) analyses. This method is the most appropriate for estimating the latent association between ordered categorical variables and will serve as a basis for evaluating item interrelationship and common factorial structure viability^51–54^.

To evaluate the construct validity of the scale, a Confirmatory Factor Analysis (CFA) model was fitted. Based on the underlying theory of RUDAS-PE, a unidimensional structure was hypothesized where the six dimensions are explained by a single latent factor of “general cognitive ability.” We used the Weighted Least Squares Mean and Variance method to estimate the model, considered the most appropriate estimator for categorical or ordinal data^51,55^. The model fit was evaluated using multiple indices: the Comparative Fit Index (CFI) and the Tucker-Lewis Index (TLI), where values > 0.90 indicate an acceptable fit and > 0.95 an excellent fit; and the Root Mean Square Error of Approximation (RMSEA), where values < 0.08 are acceptable and < 0.06 are desirable^56,57^. If the initial model did not show an adequate fit, the modification indices were examined to identify sources of misfit and guide a possible re-specification of the model, such as the removal of problematic items.

Subsequently, Item Response Theory (IRT) was applied to gain a deeper understanding of the functioning of the items and the scale as a whole. Since most dimensions of the RUDAS-PE are scored by accumulating points for correct answers (partial credit), the Generalized Partial Credit Model (GPCM) was selected. This model is ideal for polytomous items where each response category represents an incremental level of success or ability^58,59^.

### Model Fit Evaluation and Item in IRT

We used the estimation method called Marginal Maximum Likelihood and the overall fit of the GPCM model was evaluated using the M2 statistic and its associated indices (CFI, TLI, RMSEA, SRMR)^60^. At the individual level, the fit of each item to the model was examined using the S-X² statistic. This allowed us to identify whether any items behaved abnormally or inconsistently with the proposed model^61^.

Once the IRT model was adjusted and validated, the characteristic curves were generated and analyzed. The Item Characteristic Curves (ICC) graphically showed the probability of obtaining each score on an item across the entire ability spectrum^62^. The Item Information Curves (IIC) and the Test Information Curve were fundamental to the analysis. These curves indicate the ranges of the ability in which each item and the test as a whole provide the greatest measurement accuracy. The Typical Error Curve was also generated, which is inversely proportional to the information and visualizes the degree of expected error in the estimation of ability for each level of theta.

Finally, the reliability of the test was examined from the perspective of IRT, in which reliability is conceptualized as a function of ability^63^. The marginal reliability of the test, which depends on the Test Information Curve and the distribution of ability in the sample, was calculated and graphed. This approach allows us to understand how reliability varies across the ability continuum, providing a more accurate and appropriate view of measurement consistency^64^.

### Ethical considerations

This study was approved by the local community leaders (“Apus”) of each community and the ethics committees (SIDISI 215474) of the Universidad Peruana Cayetano Heredia and the Universidad Cesar Vallejo in Lima, Peru. The study was exempt from IRB review by the University of North Carolina at Chapel Hill IRB in North Carolina, USA. The study was performed in accordance with the ethical standards of the 1964 Declaration of Helsinki and its later amendments. Written informed consent was provided by each participant according to standard procedures. In the case the person was unable read and/or write, they provided their thumbprint as a proxy for signature. Participants benefited from the provision of their individual test results, as well as relevant counseling and referral to a specialist in their area when clinically indicated.

## Data Availability

Unidentifiable data included in this manuscript may be accessed upon reasonable request to the corresponding author.

## Code availability

The code utilized for this study may be accessed upon reasonable request to the corresponding author.
